# A single-cell atlas of the murine limb skeleton integrating the developmental and adult stages

**DOI:** 10.1038/s41598-025-05277-6

**Published:** 2025-07-02

**Authors:** Tim Herpelinck, Liesbeth Ory, Tom Verbraeken, Gabriele Nasello, Mojtaba Barzegari, Johanna Bolander, Frank P. Luyten, Przemko Tylzanowski, Liesbet Geris

**Affiliations:** 1 Skeletal Biology and Engineering Research Center, KU Leuven, Leuven, Belgium; 2 Prometheus, Division of Skeletal Tissue Engineering, KU Leuven, Leuven, Belgium; 3 Biomechanics Section, Department of Mechanical Engineering, KU Leuven, Leuven, Belgium; 4 Wake Forest Institute for Regenerative Medicine, Wake Forest School of Medicine, Winston-Salem, 7 NC USA; 5 Department of Biomedical Sciences, Laboratory of Molecular Genetics, Medical University of Lublin, Lublin, Poland; 6 Biomechanics Research Unit, GIGA In Silico Medicine, University of Liège, Liège, Belgium

**Keywords:** Atlas, Single-cell, Transcriptome, Limb, Morphogenesis, Bone, Bioinformatics, Bone development, Bioinformatics, Transcriptomics, Data processing, Genetic databases, Cartilage development, Limb development

## Abstract

The recent growth of single-cell transcriptomics has made single-cell RNA sequencing (scRNA-seq) into a near-routine technique. Breakthroughs in scalability have led to the creation of organism-wide transcriptomic datasets, aiming to comprehensively profile the cell types and states within an organism throughout its lifecycle. However, the skeleton remains an underrepresented organ system in organism-wide atlases. Given the skeleton’s critical role as the central framework of the vertebrate body, its function in housing the hematopoietic niche, and its involvement in metabolic and homeostatic processes, its underrepresentation presents a significant gap in current reference atlas projects. To address this issue, we integrated ten separate murine, publicly available scRNA-seq datasets, which include limb skeletal cells and their developmental precursors, resulting in an atlas of 133,332 cells. This limb skeletal cell atlas describes cells within the mesenchymal lineage, focusing on the process from limb induction to adult bone formation, and encompasses 39 well-characterized cell types and states. By expanding the repertoire of time points and cell types within a single dataset, we enable more complete analyses of cell-cell communication or in silico perturbation studies. Together, these efforts present a valuable resource for researchers in skeletal biology, metabolism, and regenerative medicine, filling an important gap in current atlas mapping projects.

**Supplementary Information:**

The online version contains supplementary material available at 10.1038/s41598-025-05277-6.

## Introduction

The skeleton is a highly advanced organ with a wide variety of functions, ranging from protection of the internal organs and supporting locomotion to calcium homeostasis, housing the hematopoietic system and serving an endocrine function^1^. In addition, some skeletal tissues possess remarkable regenerative properties, with bone being able to spontaneously regenerate after fracture with minimal scar formation^2^. While complex in its functions, the skeletal system is composed of a relatively limited set of core cell types, particularly within the structural and regulatory compartments of bone tissue, such as osteoblasts, osteocytes, chondrocytes, bone lining cells, and osteoclasts^3^. Its functional diversity appears to arise from a high degree of intrinsic heterogeneity within these cell types, allowing for regionalized specialization across skeletal sites. In contrast, non-mesenchymal compartments such as the bone marrow, harbor a much broader variety of hematopoietic and immune cell types^4^.

Advancements in ultra-high-throughput single-cell RNA sequencing (scRNA-seq) technologies, along with the development of computational algorithms to analyze the data, enable the creation of organism-wide transcriptome maps that are resolved in both time and space^5–8^. To provide a complete representation of a tissue, atlases must account for biological variability, including multiple models, strains, and diverse environmental conditions. This approach enhances the generalizability of atlas-based findings^9^. Several high-profile efforts, such as the Tabula Muris, Mouse Cell Atlas, and Human Cell Atlas, have successfully applied this principle, generating comprehensive references by integrating data across tissues, developmental stages, and experimental conditions. These resources show how harmonized datasets can support automatic annotation and consistent classification across studies^10–13^.

However, the skeletal system remains underrepresented in most existing atlases, often lacking detailed annotation of skeletal lineages or user-friendly methods for interpretation. This results in a significant gap in the transcriptional characterization of the skeletal system. While large atlases contain abundant data, much of it remains unexplored due to imprecise annotations, whereas specialized datasets remain confined within their specific research domains. To address this gap, we reannotated and integrated publicly available datasets focused on murine mesenchymal and skeletal cell types and states. These datasets span various sexes, strains, and anatomical locations, allowing us to incorporate the natural biological variability within the skeletal system. Integrating this diversity is essential to create an atlas that reflects the full spectrum of skeletal cell phenotypes and is generalizable across experimental contexts. By combining these datasets into a single reference framework of 133,332 cells, we provide a more robust and inclusive foundation for skeletal cell classification. We ensured consistency across all datasets by using the original labels and enhancing them with more detailed annotations through the use of marker genes, creating the Limb Skeletal Cell Atlas (LSCA).

**Fig. 1:**
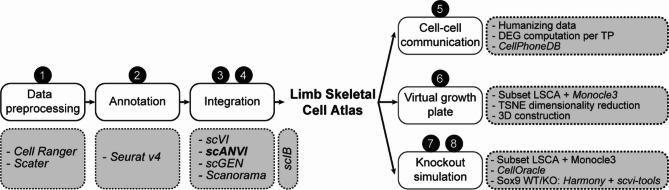
Flow diagram of computation methods. (1) Publicly available datasets were preprocessed using Cell Ranger and Scater. (2) Individual datasets were then clustered and annotated using Seurat v4. (3) We tested scVI, scANVI, scGen and Scanorama and (4) evaluated them with the scIB benchmarking package. scANVI was used to create the final Limb Skeletal Cell Atlas. (5) Cell-cell interactions for early limb bud signaling pathways were predicted using CellPhoneDB and (6) the growth plate was reconstructed with the use of Monocle3 pseudotemporal ordering. (7) Finally, an in silico knockout simulation for Sox9 was performed with Monocle3 and CellOracle and (8) evaluated with in vivo wild-type and knockout data integrated/projected with Harmony and scvi-tools. *WT* wild-type, *KO* knockout

Following the generation of the LSCA, we explored its applicability and predictive value, as schematically represented in Fig. 1. We demonstrated the potential of the LSCA in intercellular communication analysis and pseudotemporal trajectory inference. The predictive capacity of the LSCA was further tested by simulation of SRY-box transcription factor 9 (Sox9) inactivation and the analysis of its consequences, which were in line with the in vivo phenotype^14^. Collectively, these results support the notion that the LSCA can be used, amongst others, as a reliable reference for the automated annotation of new skeletal scRNA-seq datasets.The name for the Atlas was chosen to emphasize the focus on mesenchymal and skeletal tissues of the limb while also including some, but not all, other cell types such as muscle, endothelial and hematopoietic lineages. To facilitate accessibility and reproducibility, all notebooks required to analyze the datasets, build the atlas and perform the subsequent analyses have been made available online.

## Results

### Dataset annotation and integration to produce a limb skeletal cell atlas

To build a reference atlas of the limb skeleton, we selected ten publicly available mouse scRNA-seq datasets containing cells from the onset of limb development to mature bone^14–23^. These datasets encompass a broad range of developmental stages (E10.5 to 16 weeks), anatomical sites (forelimb, hindlimb, femur, tibia, humerus, cortical bone, periosteum, endosteum, bone marrow, and synovium), and skeletal compartments (growth plate, epiphysis, bone marrow stroma). The majority of the datasets were derived from C57BL/6 strains, with some datasets also including transgenic reporter lines such as Osx-Cre: GFP and CTSK-mGFP (Sup. Table 1). A comprehensive analysis was conducted on a total of 133 332 individual cells that met the quality control filters. The individual datasets were manually reannotated based on the original labels and canonical marker gene expression (Sup. Figure 1, Sup. Table 2). Next, we selected four top-performing methods from the benchmarking study by Luecken et al. to determine the most suitable integration approach for our data^24^. We tested both an unsupervised (single-cell Variational Inference; scVI)^25,26^ and a semi-supervised (single-cell Annotation using Variational Inference; scANVI)^25,27–30^ integration model, selected based on their consistent performance in complex integration tasks^24^. The scVI model allowed us to infer the underlying structure of the data without prior labels, while scANVI leveraged both labeled and unlabeled data to improve annotation accuracy. Additionally, we evaluated Scanorama, which uses a method similar to computer vision algorithms for panorama stitching, identifying and merging images with overlapping content into a cohesive panorama^31^. We also considered scGen, a generative model designed with a variational autoencoder architecture to achieve near-linear mappings for non-linear sources of variation, particularly useful for correcting batch effects using labeled data^32^.

**Fig. 2:**
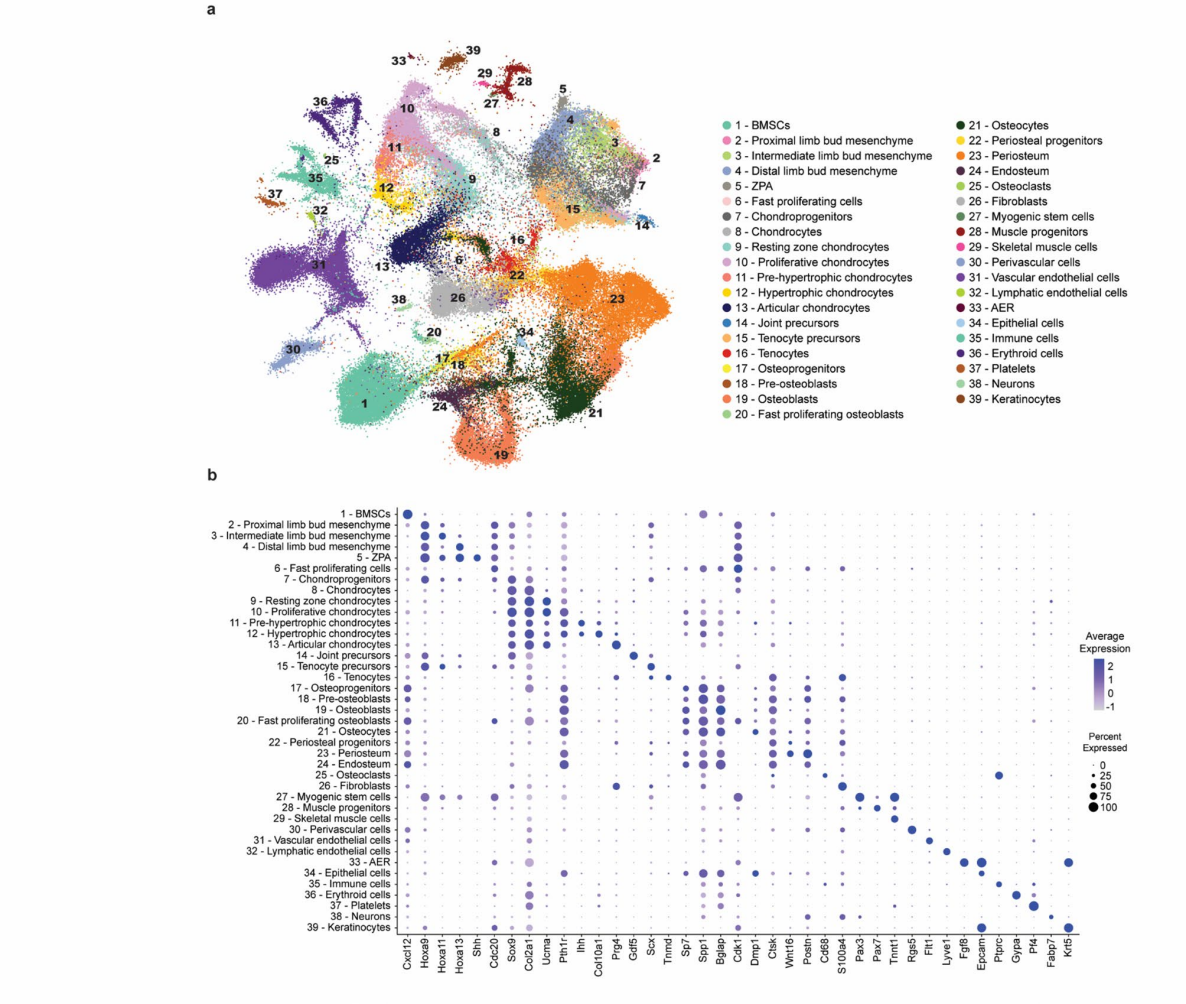
An integrated compendium of skeletal cell types with detailed annotation. **a** UMAP visualization of the scANVI latent space of 133 332 murine limb mesenchyme- and skeleton-derived cells, colored by annotation. **b** Dot plot showing the expression of one selected marker gene per cluster. The color of the dot represents the mean expression level and its size represents the percentage of cells within the cluster in which that gene was detected. For visual clarity, a single representative gene was chosen per cell type, prioritizing specificity over abundance. This visualization is intended to illustrate the distinctiveness of each cluster. Full marker combinations used for annotation are provided in Supplementary Table 2. *BMSCs* bone marrow-derived stromal cells, *ZPA* zone of polarizing activity, *AER* apical ectodermal ridge

To compare the effectiveness of these integration methods, we employed metrics focused on batch correction while preserving the biological variability inherently present in the data (scIB)^24,25^ (Sup. Figure 2). scANVI demonstrated a more favorable performance, achieving the highest label isolation score and effectively integrating the datasets without introducing cross-laboratory batch effects (Sup. Figure 3). The robust performance of scANVI ensured that the integration was cell-based, accurately representing 39 distinct cell states (Fig. 2a, b). Given the complexity of our data, this integration approach provides a reliable foundation for downstream analyses and applications. Twenty-six clusters originated from a mesenchymal lineage (Clusters #1–26) and encompassed mesenchyme, chondrocyte, osteoblast/osteocyte or fibroblast cell states. A further four states were associated with the muscle lineage (Clusters #27–30) and other clusters included endothelial (Clusters #31–32), epithelial (Clusters #33–34), hematopoietic (Clusters #35–37), neuronal (Cluster #38) and ectodermal (Cluster #39) cell states (Fig. 2a, b).

One of the drawbacks of single-cell RNA sequencing is the loss of spatial data. However, a pseudospatial context within the cells of the early limb bud can be reconstructed by identifying distinct gene expression patterns. For this purpose, we defined the proximal-distal axis by mapping the expression of the Hoxa/d9-13 mRNAs, from the most proximal (Hoxd9) to the most distal (Hoxd13) (Fig. 2b)^33^. The expression of Sonic Hedgehog (Shh) mRNA was used to define the Zone of Polarizing Activity (ZPA) located at the posterior part of the limb bud^34^. A subset of ectodermal cells situated at the distal tip of the limb bud, called the Apical Ectodermal Ridge (AER), is a signaling center associated with proximal-distal limb growth. It could be identified by the expression of Fibroblast growth factor 8 (Fgf8) mRNA^35^.

To validate the cluster annotations in the LSCA, we compared the differentially expressed genes of specific cell populations—AER, distal mesenchyme and hypertrophic chondrocytes—with published experimental data. We focused on genes that are not widely recognized as traditional markers for limb development but nevertheless exhibited distinct expression patterns within the clusters. For each cluster, we identified a key gene: Sp8^36^ for the AER, Hottip^37^ for the distal mesenchyme and Loxl4^38^ for the hypertrophic chondrocytes in the growth plate (Sup. Figure 4). We then confirmed their expression in the corresponding tissues by consulting independent (whole mount) in situ hybridization data from the literature. While these genes are not established markers in the field, their specific expression patterns in the literature provided additional support for our cluster annotations and highlighted novel molecular features within these cell populations, further validating the LSCA.

### Intercellular communication inference across the developing limb

**Fig. 3:**
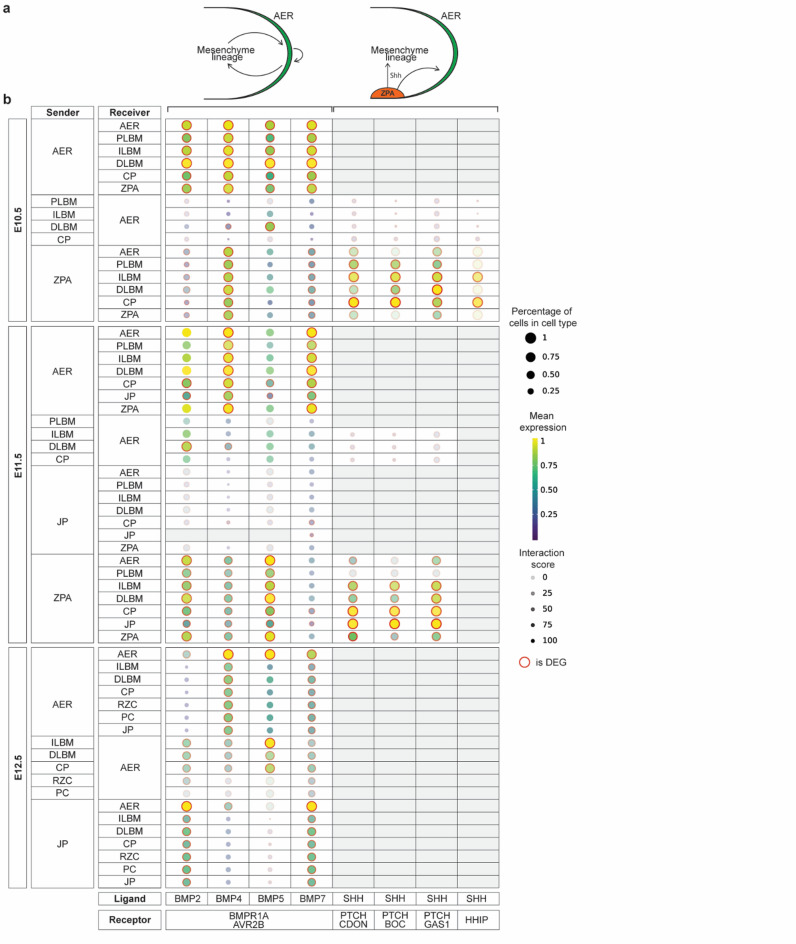
BMP and SHH signaling case study between AER, ZPA and mesenchyme. **a** Schematic illustration for visualizing AER and ZPA signaling. **b** Predicted ligand-receptor interactions across developmental timepoints (E10.5, E11.5 and E12.5). The dot plot illustrates ligand-receptor interactions between sender and receiver cell types (y-axis) and ligand-receptor pairs (x-axis). Dot color indicates mean gene expression, while dot size represents the proportion of cells within each cell type expressing the gene. Translucency reflects interaction specificity and differentially expressed genes are marked with an outer red ring. *DEG* differentially expressed genes, *AER* apical ectodermal ridge, *PLBM* proximal limb bud mesenchyme, *ILBM* intermediate limb bud mesenchyme, *DLBM* distal limb bud mesenchyme, *CP* chondroprogenitors, *RZC* resting zone chondrocytes, *PC* proliferative chondrocytes, *PHC* pre-hypertrophic chondrocytes, *JP* joint precursors, *ZPA* zone of polarizing activity

Intercellular communication driven by ligand-receptor interactions is one of the mechanisms regulating cellular differentiation. Therefore, a wide variety of tools to infer intercellular signaling from scRNA-seq data has been developed^39^. Here, we used CellPhoneDB^40^ as it considers multimeric receptor complexes when inferring ligand-receptor interactions. We analyzed Bone Morphogenetic Protein (Bmp) and Shh signaling taking place within the limb bud, the AER and the ZPA (Fig. 3a).

Bmps are members of the Transforming Growth Factor (Tgf) ligand superfamily and signal through a tetrameric receptor complex. Type I and Type II Bmp receptors are expressed across the limb mesenchyme^41,42^ and AER^41^which also acts as a source of Bmps^43^. However, due to the multimeric character of the receptor complexes resulting in many different combinations, it is challenging to experimentally determine which ligand-receptor pair is predominantly used. Screening with CellPhoneDB narrowed down the number of possible combinations. The analysis revealed that the AER mainly signals to the distal limb bud mesenchyme through Bmp4 and Bmp7, particularly at E11.5 (Fig. 3b, Sup. Figure 5–7) and that they appear to favor binding a receptor complex containing Bmpr1a. The regulation of the AER by Bmp signaling, whether from the AER itself, the mesenchyme, or both, remains a topic of ongoing discussion in the field^43^. Our analysis indicates that autocrine signaling is the primary mediator, particularly at E10.5. Additionally, we observed limited Bmp signaling from the limb mesenchyme towards the AER. Noteworthy is that the favored ligand-receptor combination is invariable over time (Fig. 3b, Sup. Figure 5–7).

Antero-posterior outgrowth and proximodistal polarization are interconnected to each other by a Shh epithelial-mesenchymal feedback loop^44^. Shh mRNA expression decreases as the limb bud grows, which explains the decrease in cell-cell communication from E10.5 to E11.5 and no Shh expression in E12.5. Shh signaling is regulated during limb development at many levels and one of them is a negative feedback loop involving binding of Shh to Hedgehog interacting protein (Hhip) which acts as a decoy receptor to inhibit Hedgehog signaling^45^. To demonstrate the usefulness of the LSCA, we investigated this regulatory loop and found that cell-cell communication is mostly limited to intermediate limb bud mesenchyme and chondroprogenitors (Fig. 3a, b).

### Virtual reconstruction of the growth plate

Long bones are formed through the process of endochondral bone formation. Initially, mesenchymal cells condense and then undergo a series of differentiation steps going from resting chondrocytes over proliferating to pre-hypertrophic and hypertrophic chondrocytes. These cells form columnar structures that together make up the growth plate. The hypertrophic chondrocytes then either transdifferentiate into osteoblasts or undergo apoptosis and the space is replaced by the osteoblasts producing bone tissue^46^.

**Fig. 4:**
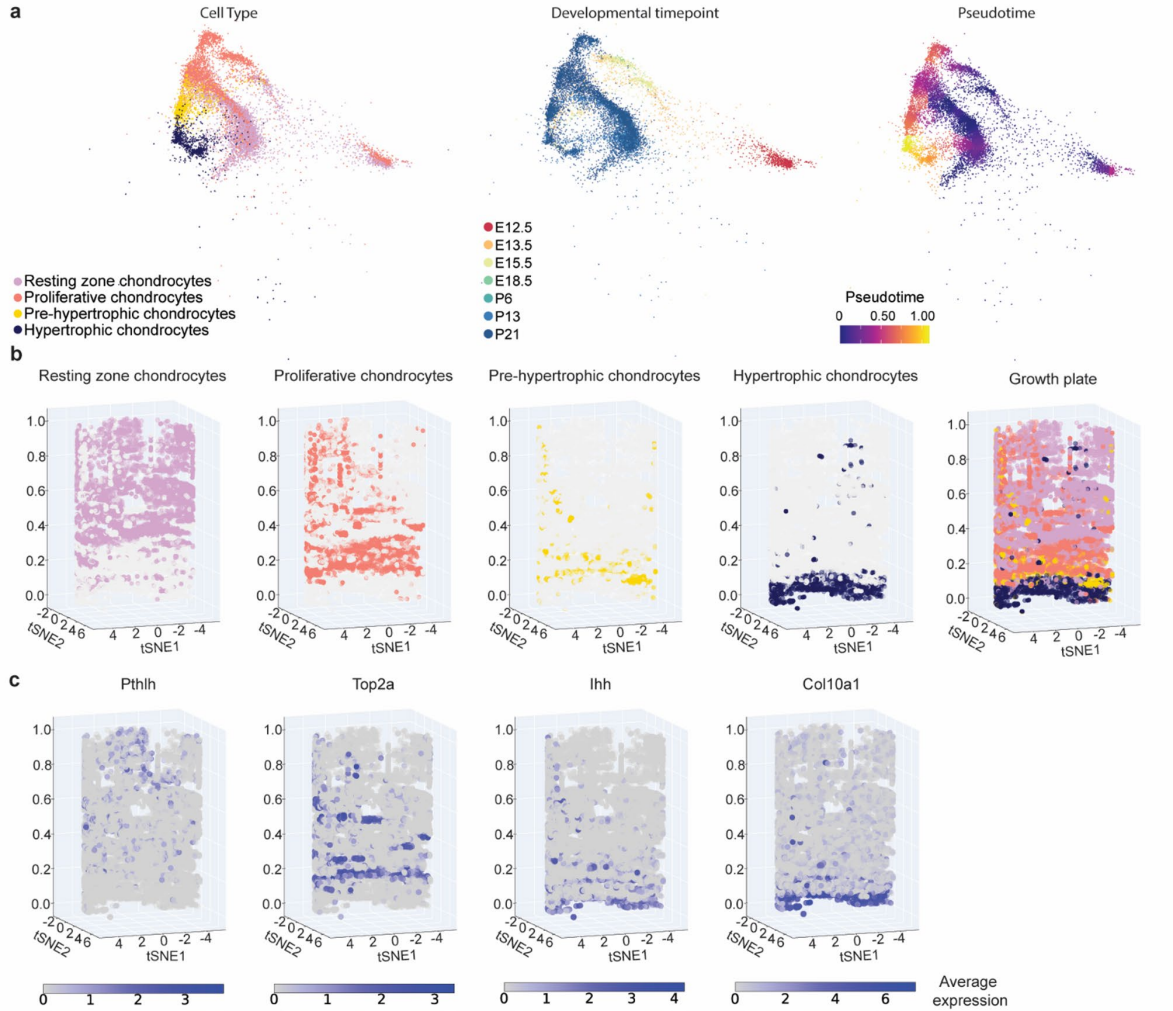
Pseudospatiotemporal reconstruction of the transcriptional dynamics within the growth plate. **a** UMAP visualization of the scANVI latent space of the integrated growth plate data subset from the main atlas, colored by annotation (left), developmental timepoint (middle) and Monocle 3 pseudotime value (right). **b** Growth plate chondrocytes were grouped in 50 bins based on similar pseudotime values. t-SNE dimensional reduction was performed on each bin, using a circle with a radius of 20 as the boundary condition for gradient descent, thus reconstructing the cylindrical shape of the growth plate upon stacking the bins. **c** Expression of known marker genes across different growth plate zones in pseudotime and pseudospatial dimensions

As a part of the validation of our atlas, we virtually restored the growth plate. To accomplish that, we used pseudotime analysis to reconstruct the transcriptional trajectories from resting to hypertrophy zone in silico (Fig. 4a, Sup. Figure 8). Cells were then binned by pseudotime and for each bin, we performed dimensionality reduction by t-distributed stochastic neighborhood embedding (t-SNE). Importantly, we imposed a circle as a boundary condition for each t-SNE. Alignment of these circular projections then recreated the cylindrical shape of the growth plate, while also visualizing transcriptional heterogeneity within the growth plate across bins of pseudotime (Fig. 4b). This approach provided an intuitive way to visualize gene expression patterns, allowing researchers, particularly those without bioinformatics expertise, to interpret the data in a 2D or 3D-like reconstruction of the growth plate. Plotting gene expression along the pseudotime axis, combined with expression in pseudospace, artificially restored to a certain extent, the tissue architecture (Fig. 4c). While this reconstruction does not provide actual spatial data, it offers an easily interpretable representation of growth plate signaling. Future iterations of the LSCA could integrate spatial transcriptomics data to provide a more precise and spatially resolved view of the growth plate.

### Simulation of transcription factor perturbation

Developmental biology has been a key field for studying gene regulatory networks (GRNs)^47^. Traditionally, this involved a series of experiments where transcription factor activity is altered by gain- or loss-of-function and the resulting in vivo effects are analyzed. With the advances in bioinformatics, the CellOracle^48^ algorithm was developed to study GRN inference from scRNA-seq data. In addition, it allows us to explore in silico the effects of the perturbation of transcription factor expression on target gene expression. To test if our dataset was amenable to this type of analysis, we decided to compare an in silico prediction to published in vivo knock-out data^14^.

Sox9 is involved in many stages of chondrogenesis, from regulating the initial mesenchymal condensation to maintaining the proliferation of columnar chondrocytes and preventing their premature transdifferentiation into osteoblasts. It also plays a crucial role in inducing chondrocyte hypertrophy, making it indispensable for the proper formation and maintenance of functional growth plates. Inactivation of Sox9 leads to a shortening of the columnar and hypertrophic zones in the growth plate and accelerates ossification due to premature pre-hypertrophy and matrix mineralization^14,49^.

**Fig. 5:**
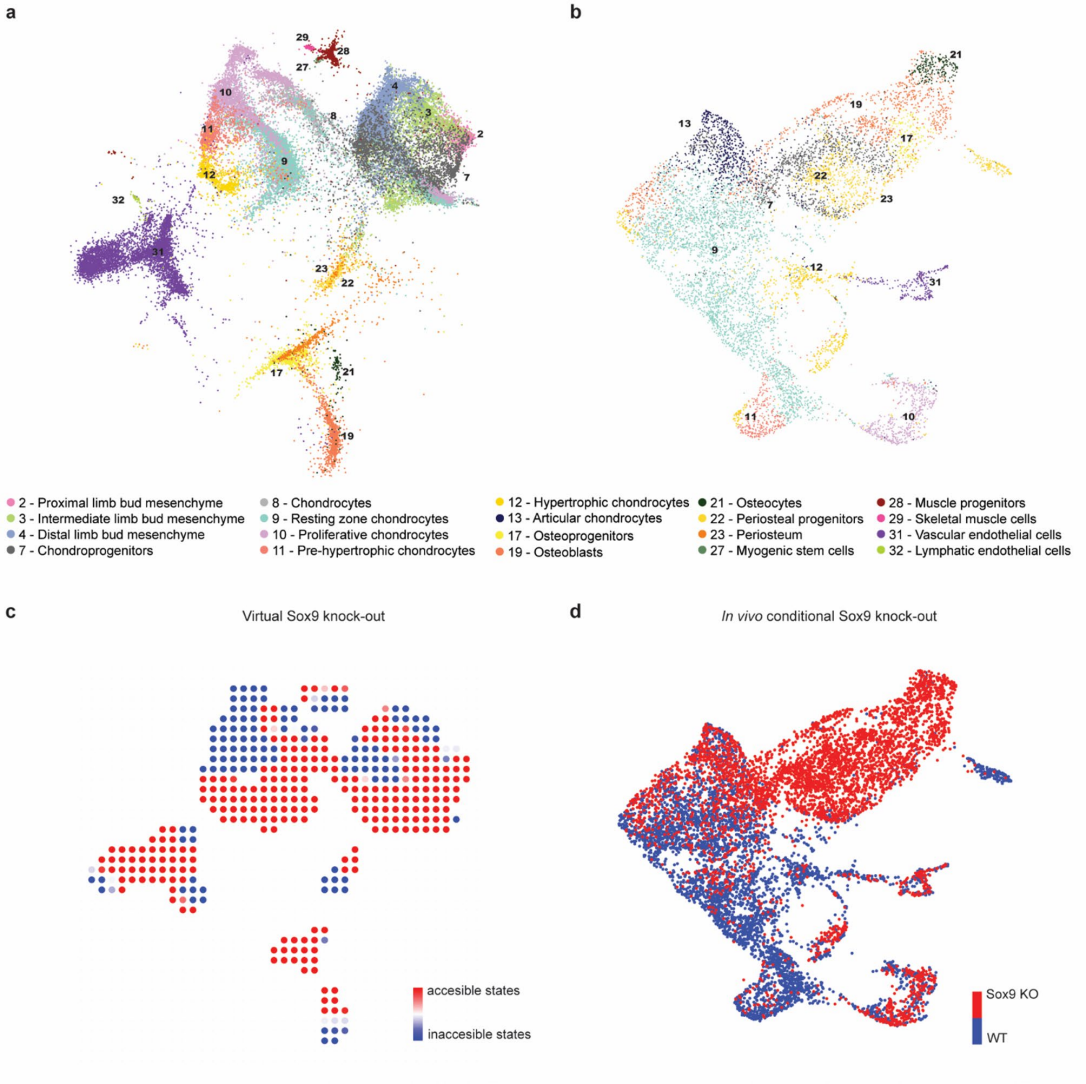
In silico predictions of Sox9 knockout (KO) compared to in vivo data. **a** Reference annotation of the subsetted atlas, which includes only the relevant cell types from developmental stages E10.5 to P21, used for knockout analysis. **b** UMAP plot integrating WT and Sox9 KO in vivo data. **c** Inner vector product of two vector fields: the pseudotime gradient from unperturbed conditions and the cell state transition probability following in silico Sox9 perturbation. Red regions indicate accessible cell states, while blue regions represent inaccessible cell states. The loss of chondroprogenitor and prehypertophic chondrocyte cell identities as accessible states reflects the biological profile of the ground truth shown in **d**. Myogenic, osteogenic and endothelial differentiation are largely unaffected. D, UMAP of integrated Sox9 KO and WT in vivo data, representing ground truth affected cell identities colored by condition (red: Sox9 KO, blue: WT)

Here, we demonstrate that the LSCA can be used to generate in silico gene knockouts, which can then be compared directly to single-cell in vivo knockout datasets and phenotypic observations, offering a robust framework to explore developmental and regulatory disruptions. To evaluate the effectiveness of the Atlas in exploring the consequences of targeted gene inactivation, we applied the CellOracle algorithm to create a virtual Sox9 knockout and compared our in silico predictions to data from an in vivo knockout study (Fig. 5a, b). For this analysis, we subsetted the LSCA to include time points between E10.5 and P21, focusing on relevant cell types such as periosteal cells, chondrocytes at various differentiation stages, osteogenic cells, endothelial cells (vascular and lymphatic), limb bud mesenchymal populations, and myogenic cells. We modeled the data of late inactivation of Sox9, driven by the Acan-Cre expressed in the growth plate. We defined the accessible cell states as those that cells can differentiate under the current regulatory conditions, while inaccessible states cannot be reached due to the absence or inactivation of essential transcription factors. Using the Atlas, we identified regions within the dataset that are either developmentally accessible or inaccessible to Sox9’s regulatory influence.

The simulations showed that upon removal of Sox9, most mesoderm-derived cells failed to contribute to cartilage formation, while myogenesis, osteogenesis, and endothelial differentiation remained largely unaffected (Fig. 5c, Sup. Figure 9, 10). Specifically, in the virtual Sox9 inactivation model, cell type 11—pre-hypertrophic chondrocytes—is represented as a blue region, indicating that mesenchymal cells cannot access this cell state. These results are supported by the in vivo findings, where Sox9 inactivation prevents mesodermal cells from differentiating into chondrocytes, leading to an accumulation of cartilage precursors and osteogenic cells (Fig. 5d). Phenotypically, the absence of Sox9 in early limb buds disrupts mesenchymal condensation, chondrocyte differentiation, and the establishment or differentiation of the osteoblast lineage^50^. This is reflected in our in silico Sox9 knockout, where the distal mesenchyme predominantly remains in an inaccessible state, indicating impaired mesenchymal condensation and chondrogenic cell fate commitment. Chondrocytes, including proliferating and pre-hypertrophic populations, as well as a substantial portion of osteoblasts are predicted to be inaccessible states, mirroring the absence of their differentiation (Fig. 5c, Sup. Figure 9, 10). Together, these findings underscore the value of the LSCA as a powerful tool for in silico modeling, enabling a deeper understanding of the mechanisms underlying gene function and their developmental consequences.

A limitation of the virtual knockout is that it accounts only for a neighborhood developmental flow but not lineage dependency. As a result, it will mark a region as an inaccessible cell state if its development depends on Sox9, however, it will mark regions dependent on this (inaccessible) cell type as accessible. For instance, cell type 12—hypertrophic chondrocytes—is shown as a red region in the virtual knockout, indicating that this cell state is predicted to be present in Sox9-perturbed situations. However, the in vivo data show that this cell type is absent in the knockout (red) because it originates from pre-hypertrophic cells.

## Discussion

We have generated a Limb Skeletal Cell Atlas (LSCA) representing a manually curated compendium of 133,332 cells across ten datasets and explored its applicability in both data- and hypothesis-driven analyses. To assemble the LSCA, we curated data from ten publicly available datasets^14–23^representing murine cells spanning various developmental stages and tissues. The integration of these datasets was carefully performed using advanced computational methods, including normalization, dimensionality reduction, clustering, and multiple integration techniques (scVI, scANVI, Scanorama, and scGen). A flow diagram summarizing the methodology used to construct the Atlas outlines each step of the preprocessing, clustering, integration, and validation processes (Fig. 1).

First, analysis of intercellular communication by Bmp signaling sheds light on a longstanding question in the field of limb development. Specifically, it is known that Bmp signaling is required for AER regression, but not whether the AER, the mesenchyme or both act as the source of those BMPs^43^. Based on CellPhoneDB’s cell-cell communication inference, considering the subunit architecture of both ligands and heteromeric receptors^39,40^. Our case study suggests the AER to be the dominant source of Bmp. This finding does, however, require further in vivo validation.

Next, we constructed the spatiotemporal map of the growth plate that recapitulated the in vivo situation. We achieved that by imposing boundary conditions on the gradient descent of t-SNE dimensionality reduction with pseudotime calculations, which revealed a progression through distinct chondrocyte subtypes. Similar results have been obtained previously by warping the principal component space^51^.

Finally, we demonstrated that the in silico inactivation of Sox9 aligns with in vivo results, showing the absence of cells dependent on Sox9 for differentiation into chondrocytes^14,52^. While these findings align with established in vivo observations, the LSCA should be considered primarily as a tool to explore hypotheses that will require further experimental validation. Given that we can assess the developmental outcome of computationally simulated transcription factor knockouts, the LSCA represents a valuable resource for both limb developmental biologists and skeletal biology researchers.

The current release is restricted to the healthy murine skeleton, with gaps in limb sites and developmental stages, as well as factors such as genetic background, sex, and the enrichment of sub-populations, which may influence the census and its downstream applications. These limitations highlight opportunities for future research to build upon our findings, addressing these gaps and expanding our understanding. We will continuously update and extend the LSCA (made available through the LSCA Github and web app, cfr infra), as data availability permits, toward the entire limb skeleton in health and disease. The goal of this study was to provide an initial reference of the skeletal system of the limb, which will serve as the basis for a collaborative effort to expand the Atlas.

While our murine LSCA offers a detailed view of limb and skeletal populations, direct comparisons to human data remain limited due to species-specific differences in development and annotation. Recent human limb single-cell atlases provide valuable opportunities for cross-species comparisons, and future work could integrate human datasets to enhance cross-species analyses and expand the LSCA’s utility for developmental and disease research^53–55^.

In our study, we employed three robust methods to validate our atlas and demonstrate its practical applications, each with its own limitations. CellPhoneDB effectively infers cell-cell interactions from single-cell RNA sequencing data, though it faces challenges with indirect protein abundance measurements and the use of different names for the same receptor or ligand. Monocle3 excels in resolving detailed pseudotemporal trajectories, although handling large, complex datasets could be better suited with alternative graph-based methods. CellOracle performs well calculating in silico gene perturbations, but a recent critique highlights its failure to account for distal regulatory interactions and a flaw that skews benchmarking scores^56^. Importantly, all data, analyses, and codes required to replicate the results are freely available via GitHub. Users can easily adjust analysis choices by modifying the provided code to incorporate alternative methods or algorithms that best fit their needs, ensuring our Atlas can be tailored to a wide range of research applications.

As a part of our effort, we created a web portal to allow browsing of the data. This interactive web-based resource makes the mRNA expression data easily accessible, allowing users to explore cell types expressing genes of interest or uncover transcriptomic subpopulations within a cell type. Cells can be filtered by cell type and by metadata from the original studies, such as sequencing technique, developmental timepoint or age, tissue origin, and study ID. Gene expression can be visualized, with options to download results and access the full atlas for further analysis.

Importantly, while the manuscript presents a fixed, representative subset of datasets to build and validate the Limb Skeletal Cell Atlas, the web portal is designed as a dynamic and continuously evolving resource. We will regularly update the portal to incorporate newly published datasets, thereby expanding the repertoire of tissues, developmental stages, and cell types represented. This ensures that the atlas remains current and increasingly comprehensive for the research community.

In short, the Limb Skeletal Cell Atlas provides a robust and flexible framework for the characterization of most known cell populations in the limb skeleton and serves as a foundation for future studies in a wide variety of disciplines.

## Materials and methods

### Data preprocessing

We selected ten publicly available scRNAseq datasets containing wild-type murine cells spanning limb and skeletal tissues during development and adulthood^14–23^. or datasets where aligned count matrices were not provided, raw sequencing files were processed using Cell Ranger to generate gene expression matrices^57^. Initial Quality control (QC) of the raw count matrices was performed independently for each dataset using the Scater package^58^. QC metrics were computed per cell, including total UMI counts (library size), the number of detected genes, and the proportion of reads mapping to mitochondrial genes. Cells were excluded if they exhibited a library size or number of detected genes deviating by more than three median absolute deviations (MADs) from the median of their respective distributions. Similarly, cells with a mitochondrial read fraction exceeding three MADs above the median were removed, as these likely represent damaged or dying cells. Genes with low abundance (expression below 1E-3) were excluded, and duplicate gene entries, if present, were removed.

### Clustering and dimensionality reduction

All datasets were analyzed individually prior to integration. The filtered count matrices were imported into Seurat v4^59^. Normalization was performed using the LogNormalize parameter and a scale factor of 1E4. Subsequently, the data was centered and scaled using all genes followed by a calculation of principal components (PCs) using the top 2000 highly variable genes selected by the “vst” method. The optimal number of PCs to construct the Shared Nearest Neighbor (SNN) graph was visually determined based on the elbow plot and varied for each dataset. Clustering was performed using the Louvain algorithm. The resolution was adapted to the individual dataset, where we defined the optimal number of clusters as the maximum number of cell states that could confidently be labeled based on marker gene expression. These marker genes were obtained from the FindMarkers function using the default settings.

### Integration with scvi-tools, scanorama and ScGen

The integrated atlas was constructed using scVI^26^ and scANVI^27–30^selected for their strong performance across complex datasets and compatibility with downstream analyses^24^. For preprocessing the datasets, we filtered for the 5000 most variable genes. First, we used scVI as an unsupervised tool to find common axes of variation between the datasets, helping to capture underlying data structures without prior labels(19). To refine the integration further, we then used scANVI, a semi-supervised tool, leveraging both labeled and unlabeled data to improve the accuracy of integration. scANVI builds on the shared axes of variation found by scVI and integrates cell type information, allowing for a data manifold that better represents the latent biological structure. The parameters were used as described in the scvi-tools^25^ tutorial of ‘*Atlas-level integration of lung data*’. For Scanorama, we used parameters from the step-by-step tutorial provided by the National Bioinformatics Infrastructure Sweden (NBIS), as described on the Scanorama GitHub page^31^. We used the scGen batch removal tutorial with standard parameters from the scGen documentation page (readthedocs)^32^.

### Integration metrics

To calculate integration metrics, the scIB-metrics package was used^24^which contains scalable implementations of the metrics used in the scIB benchmarking suite^60–63^. Bio conservation was captured with the use of classical label conservation metrics, which assess local neighborhoods (graph cLISI, extended from cLISI), global cluster matching (Adjusted Rand Index: ARI, normalized mutual information: NMI) and a metric evaluating rare cell identity annotations (isolated label scores). Batch correction scores were measured via the k-nearest-neighbor batch effect test (kBET), k-nearest-neighbor (kNN) graph connectivity and the average silhouette width (ASW) across batches. Independently of cell identity labels, batch removal is measured using the graph integration local inverse Simpson’s Index (graph iLISI, extended from iLISI) and PCA regression.

### Validation of differentially expressed genes

To validate the cell annotation and evaluate the potential of the LSCA for novel marker gene detection, differential expression analysis was performed for all cell identities, retaining the top 10 results returned by the *FindMarkers()* function. As these genes are statistically the most specific for their respective cell state, they represent prime candidate biomarkers. Reassuringly, known canonical marker genes were present for each annotation. We then focused on the DEG lists of the Apical Ectodermal Ridge (AER), distal limb mesenchyme, and hypertrophic chondrocyte clusters. An extensive literature search was performed for the expression patterns of DEGs not considered canonical markers for these cell types. Based on this search, we were able to confirm the specific expression of Sp8 in the AER, Hottip in the distal mesenchyme and Loxl4 in hypertrophic chondrocytes as shown by (whole mount) in situ hybridization experiments on mouse embryos (Supp. Figure 4). These genes showed clear and specific expression patterns, supporting the accuracy of the cluster.

### Cell-cell interaction prediction

Prediction of cell-cell communication by ligand-receptor interactions between cell types was performed using CellPhoneDB v5^40^. CellphoneDB is an open-access resource that provides a curated collection of receptors, ligands, and their interactions. The atlas was subsetted for each developmental time point before downstream cell-cell communication analysis. For each subset, the translate function was used to humanize the data and normalized. Differentially expressed genes were computed for each cell type and used as input for ligand-receptor pair calculations.

### Virtual growth plate reconstruction

We first subsetted the atlas for growth plate chondrocytes: Resting, Proliferative, Pre-hypertrophic and Hypertrophic chondrocytes. This subset within the latent space of the scANVI integration was then passed to Monocle 3^64^. Clustering was performed on the resulting CellDataSet (CDS) object using the default parameters. The trajectory graph was learned on the monocle-derived clusters by calling learn_graph. Roots were manually chosen with order_cells. The resulting pseudotime was added to the metadata of the growth plate subset within the latent space of scANVI and used as input for the growth plate reconstruction. Cells were binned by pseudotime using the cut function from- pandas^65,66^. Upon each bin, we performed dimensionality reduction by t-distributed stochastic neighborhood embedding (t-SNE). We imposed a circle as a boundary condition on the gradient descent function of each t-SNE. With the concatenation function, we were able to stack the circular projections in an ordered way to recreate the cylindrical shape of the growth plate.

### Knockout simulation

For in silico knockout experiments in CellOracle^48^ the atlas was subsetted to only include time points E10.5-P21 and relevant cell types (Periosteal progenitors, Periosteum, Endosteum, Resting zone chondrocytes, Proliferative chondrocytes, Pre-hypertrophic chondrocytes, Hypertrophic chondrocytes, Chondrocytes, Chondroprogenitors, Osteoprogenitors, Pre-osteoblasts, Osteoblasts, Osteocytes, Vascular endothelial cells, Lymphatic endothelial cells, Proximal limb bud mesenchyme, Intermediate limb bud mesenchyme, Distal limb bud mesenchyme, Myogenic stem cells, Muscle progenitors and Skeletal muscle cell). This subset within the latent space of the scANVI integration of the atlas was then passed to Monocle 3^64^. Clustering was performed on the resulting CellDataSet (CDS) object using the default parameters. The trajectory graph was learned on the monocle-derived clusters by calling learn graph. Roots were manually chosen with order cells. The resulting pseudotime was added to the metadata of the knockout subset within the latent space of scANVI and used as input for the virtual knockout. To reduce the amount of computational time and resources required by a large dataset, 20,000 cells were randomly selected and only highly variable genes (*n* = 5000) were included. For gene regulatory network (GRN) inference, we used the built-in base GRN made from the mouse sci-ATAC-seq atlas^67^ CellOracle offers several pre-built base GRN options and provides pipelines to create a custom base GRN using your own scATAC-seq data. For mouse analyses, they recommend using the GRN built from the mouse sciATAC-seq Atlas dataset, which includes a variety of tissues and cell types. Following k nearest neighbors (KNN) imputation based on the first 27 PCs, GRNs were imputed for each cluster. To simulate the knockout of a transcription factor, its expression was set to 0. After this knockout, GRN inference was performed again. Signal perturbation propagation and transition probabilities were calculated using the standard settings. Visualization of the pseudotime gradient, simulation vector field and their inner product was performed as described in the CellOracle online documentation.

### In vivo Sox9 knockout and WT data analysis and integration

Both wild-type (WT) and Sox9 knockout (KO) dataset of Haseeb et al.^14^were preprocessed separately to ensure high-quality data for subsequent analyses. The preprocessing steps included quality control measures to remove low-quality cells, as described in “Data preprocessing”. Following preprocessing, cells from both WT and Sox9 KO datasets were clustered and labeled independently. Clustering was conducted to identify distinct cell populations within each condition, and labels were assigned based on known cell markers, as described in “Clustering and dimensionality reduction”.

To integrate the WT and Sox9 KO datasets, the Harmony package^60^ was employed. Harmony facilitates the integration of multiple datasets by correcting batch effects and aligning data across different conditions. This integration enabled a comprehensive comparison of cell populations and gene expression profiles between WT and Sox9 KO conditions. Harmony is particularly suited for integrating datasets from the same study with well-defined batch and biological structures, making it ideal for this analysis^24^.

As an additional validation step, we employed reference mapping using scANVI to align the in vivo data with the reference latent space of the Atlas (Sup. Figure 9). We followed the *‘Reference mapping with SCANVI’* tutorial, utilizing the scANVI model trained for integration to predict cell type labels^25,30^.

## Electronic supplementary material

Below is the link to the electronic supplementary material.

Supplementary Material 1

## Data Availability

The datasets analyzed for this study can be found in Sup. Table 1. The Limb Skeletal Cell Atlas can be downloaded or interactively explored at www.skeletalcellatlas.org. All code used to perform the analyses and notebooks to generate the figures is available at https://github.com/TElabSBE/LimbSkeletalCellAtlas.
