# Supplementary material for: A single-cell atlas of the murine limb skeleton integrating the developmental and adult stages
Source: Sci Rep. 2025 Jul 2;15:22514. doi: 10.1038/s41598-025-05277-6 (PMC12215971; doi:10.1038/s41598-025-05277-6)
Supplement: Supplementary file 1 — Supplementary Material 1 [file 41598_2025_5277_MOESM1_ESM.docx]

# A single-cell atlas of the murine limb skeleton integrating the developmental and adult stages

# Supplementary Material


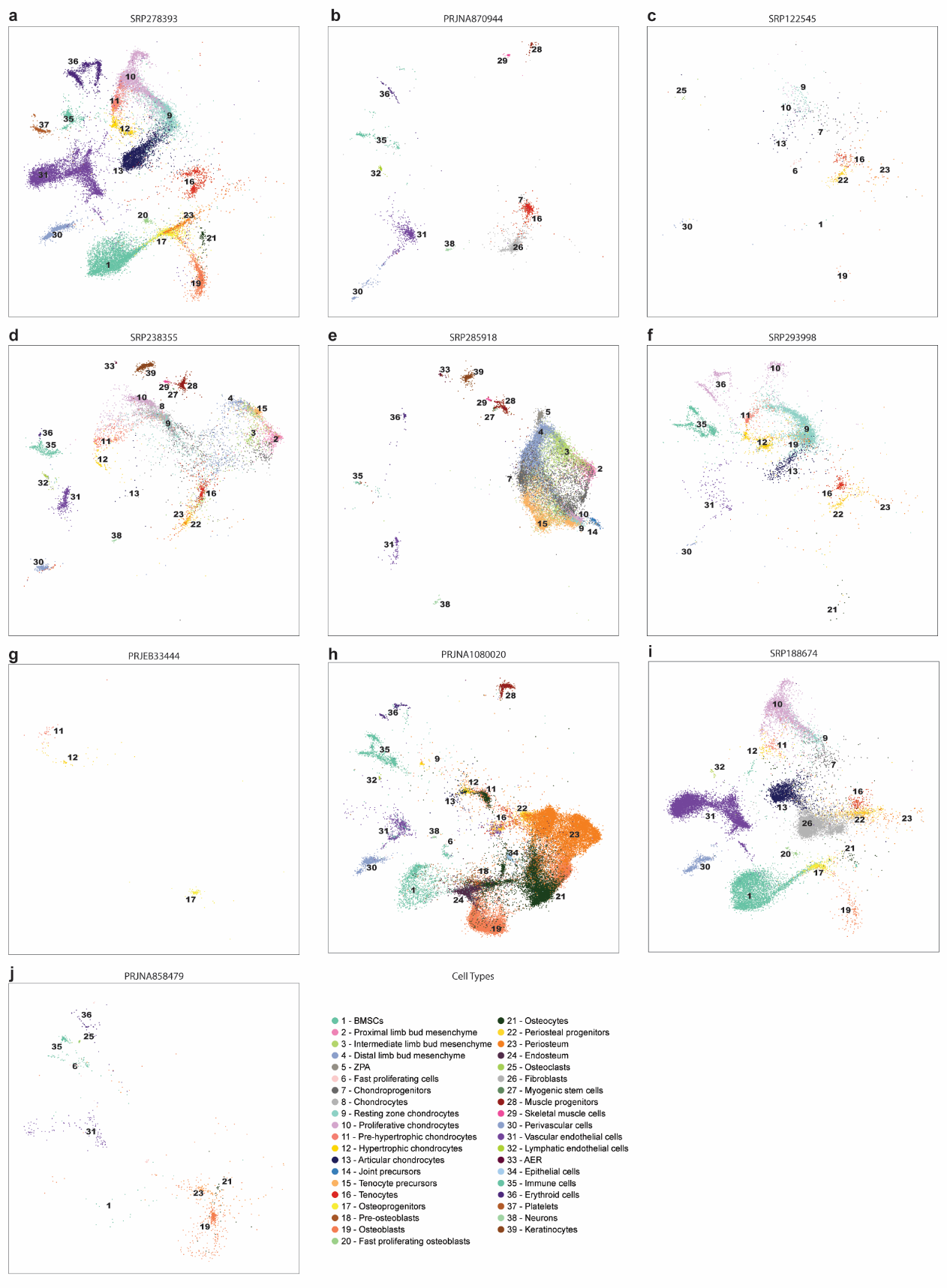


**Supplementary Figure 1. Individual datasets after reannotation**. **a-j**, UMAP visualization of the scANVI latent space, colored by cell type, for each of the 10 separate datasets. Each panel represents a distinct dataset, identified by its database ID: **a**, 34260921 **b**, 36175067, 37873464 **c**, 30250253 **d**, 31874220 **e**, 33297480 **f**, 33597301 **g**, 31543445 **h**, 38479598 **i**, 31130381 **j**, 36777346. These visualizations highlight the distribution of different cell types after reannotation, offering insights into the data structure and heterogeneity across datasets.

**
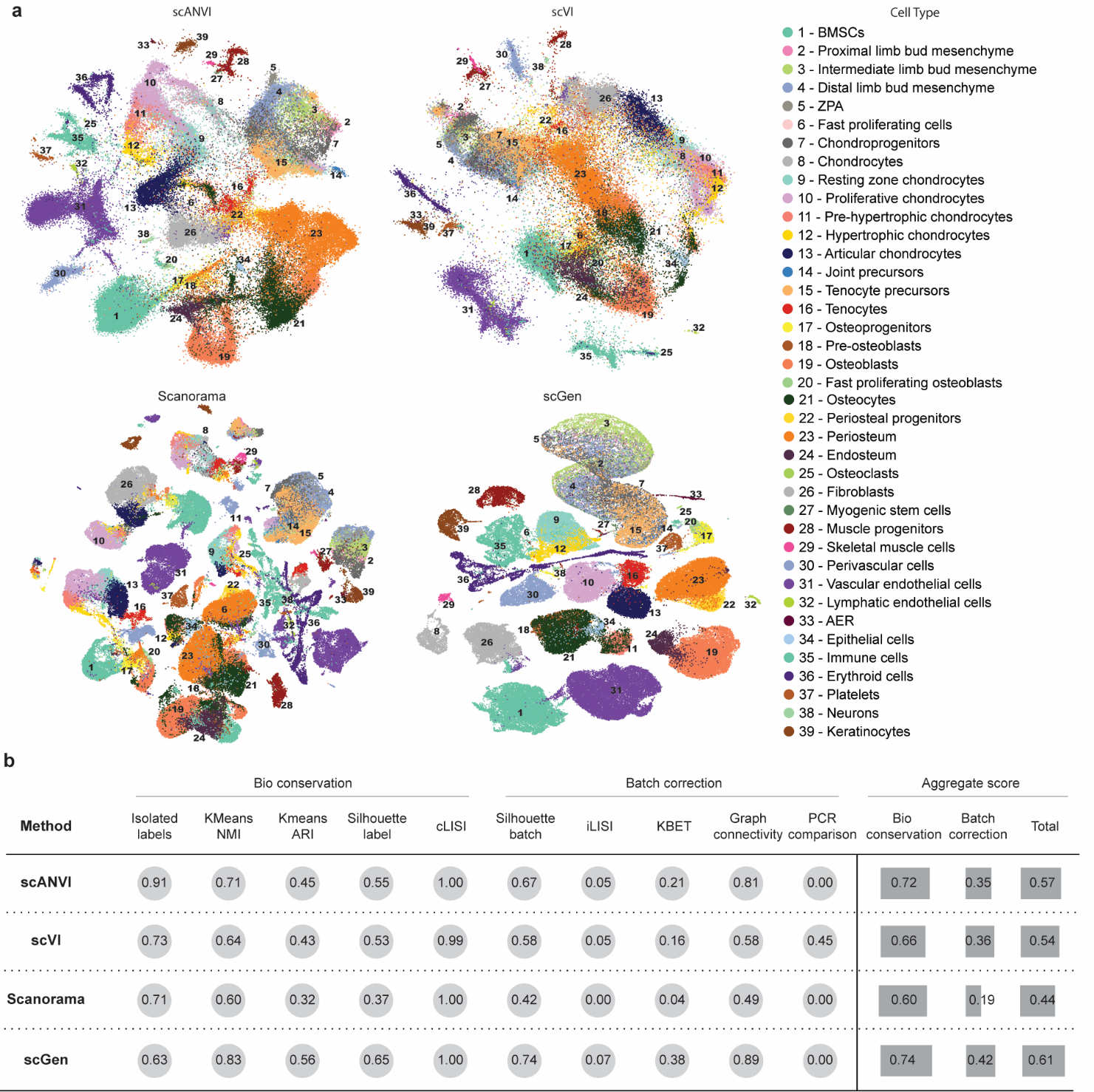
**

**Supplementary Figure 2. Assessment of integration method**. **a**, UMAP visualization comparing the latent spaces generated by different integration methods: scANVI, scVI, Scanorma, and scGen. Each method's latent space is visualized separately to assess how well the datasets integrate across methods. **b**, Results of the integration quality assessment using scIB. Biological conservation metrics quantify the integrity of cluster-based metrics of the integration output. Each value is scaled from 0 to 1, where larger scores represent better conservation of the biological aspect addressed by that metric. Batch correction metrics values are scaled between 0 and 1, in which larger scores represent better batch removal. Aggregate scores for bio conservation and batch correction are the mean values of their respective columns. The total integration score (“Total”) is calculated as a weighted average of batch correction (40%) and biological conservation (60%) metrics. ARI: adjusted Rand index, NMI: normalized mutual information, cLISI, cell-type local inverse Simpon’s index (LISI) score, iLISI: integration LISI score, KBET: *k*-nearest neighbor graph effect test, PCR: principal component regression score.


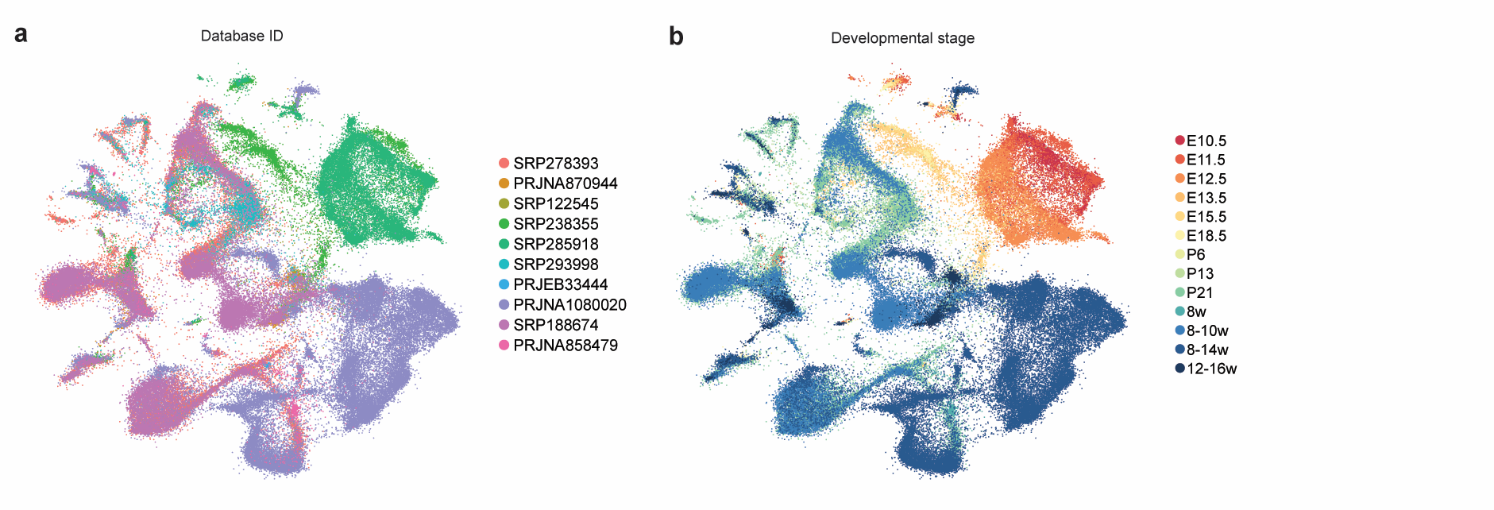


**Supplementary Figure 3. Assessment of batch effects influencing integration.** **a**, UMAP visualization of the scANVI latent space colored by study, showing the distribution of datasets. **b**, UMAP visualization of the scANVI latent space colored by developmental stage or age, illustrating how the integration reflects these biological variables.


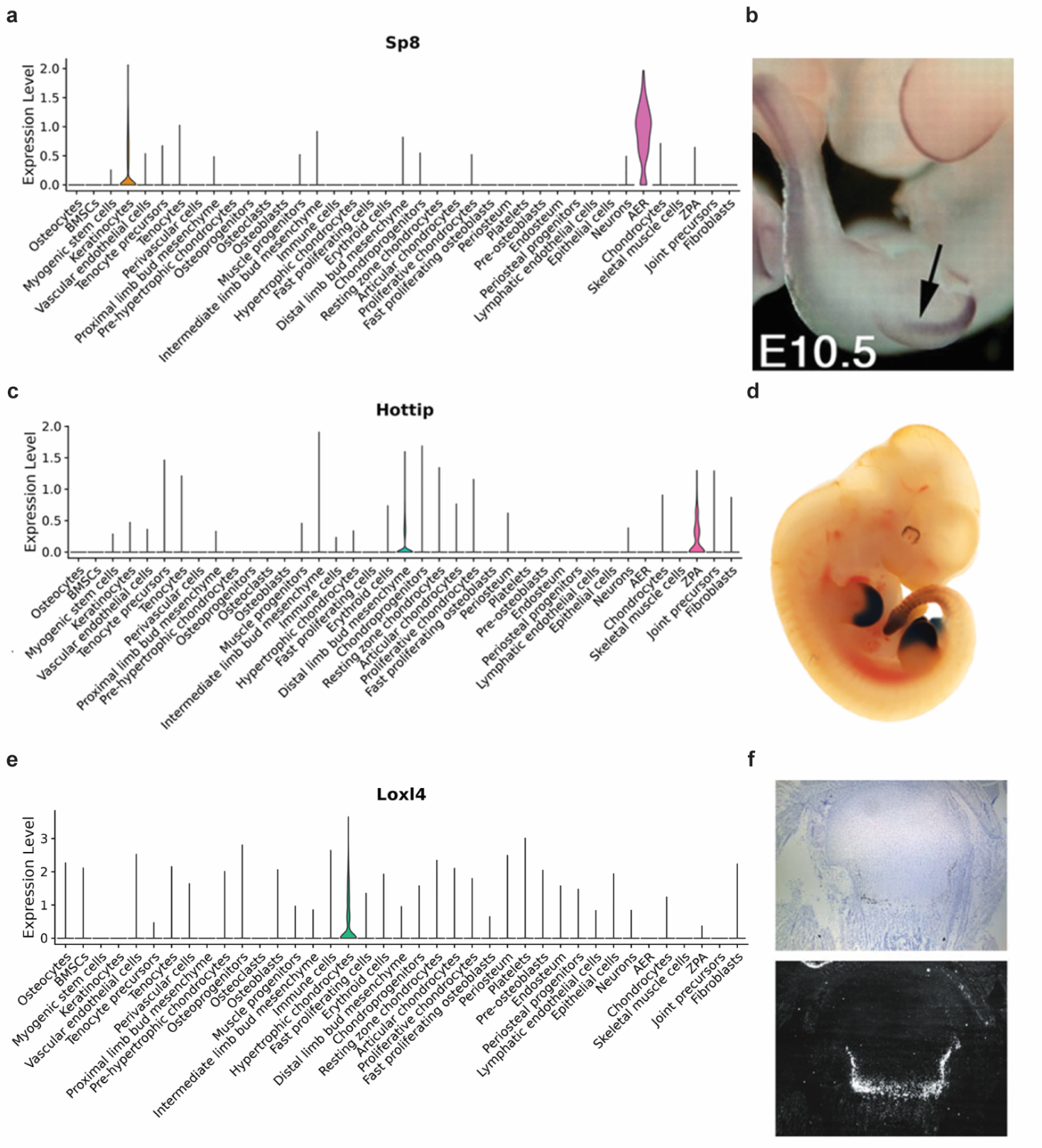


**Supplementary Figure 4. Validation of differentially expressed genes.** **a-f**, Single-cell violin plots showing the expression of Sp8 (**a**), Hottip (**c**), and Loxl4 (**e**), alongside supporting whole mount in situ hybridization data for each gene. The *in situ* data for Sp8 is adapted from Bell, S. et al., *Development* 135, (2008) (**b**); Hottip from Lai, KM. et al., *PLoS One* 10, (2015) (**d**); and Loxl4 from Ito, H. et al., *J Biol Chem*. 26, (2001) (**f**).


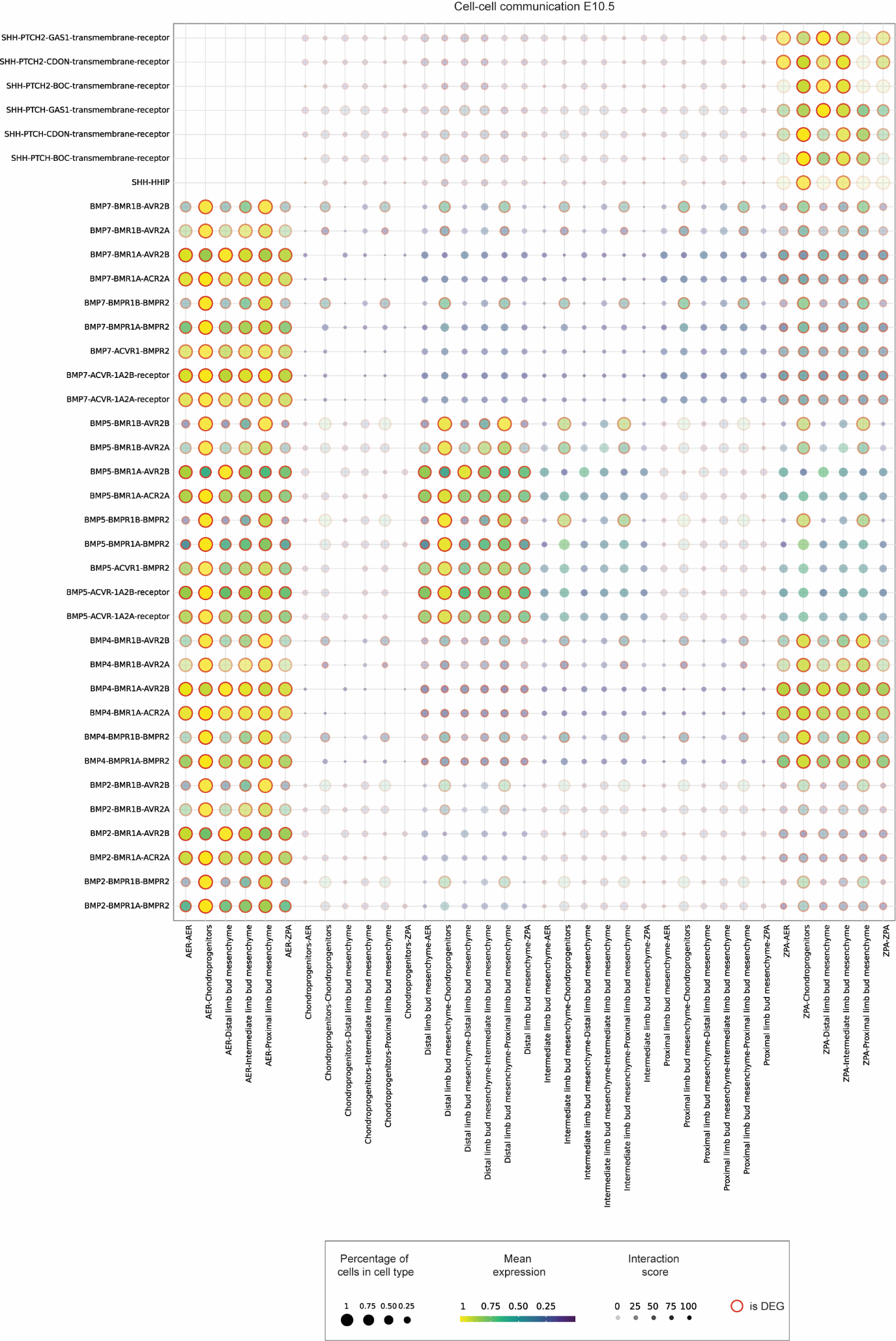


**Supplementary Figure 5. BMP, GDF5 and SHH signaling case study between AER, ZPA and mesenchyme for developmental timepoint E10.5.** Dot plot showing predicted ligand-receptor complex interactions at E10.5. The sender and receiver cell types are displayed on the x-axis, while ligand/receptor pairs are shown on the y-axis. The color intensity of each dot represents the mean gene expression, and the size indicates the percentage of cells expressing the gene within each cell type. Translucency reflects interaction specificity, and an outer red ring indicates differentially expressed genes (DEGs). Cell types: AER: Apical Ectodermal Ridge, PLBM: Proximal limb bud mesenchyme, ILBM: Intermediate limb bud mesenchyme, DLBM: Distal limb bud mesenchyme, CP: Chondroprogenitors, RZC: Resting zone chondrocytes, PC: Proliferative chondrocytes, PHC: Pre-hypertrophic chondrocytes, ZPA: Zone of Polarizing Activity.


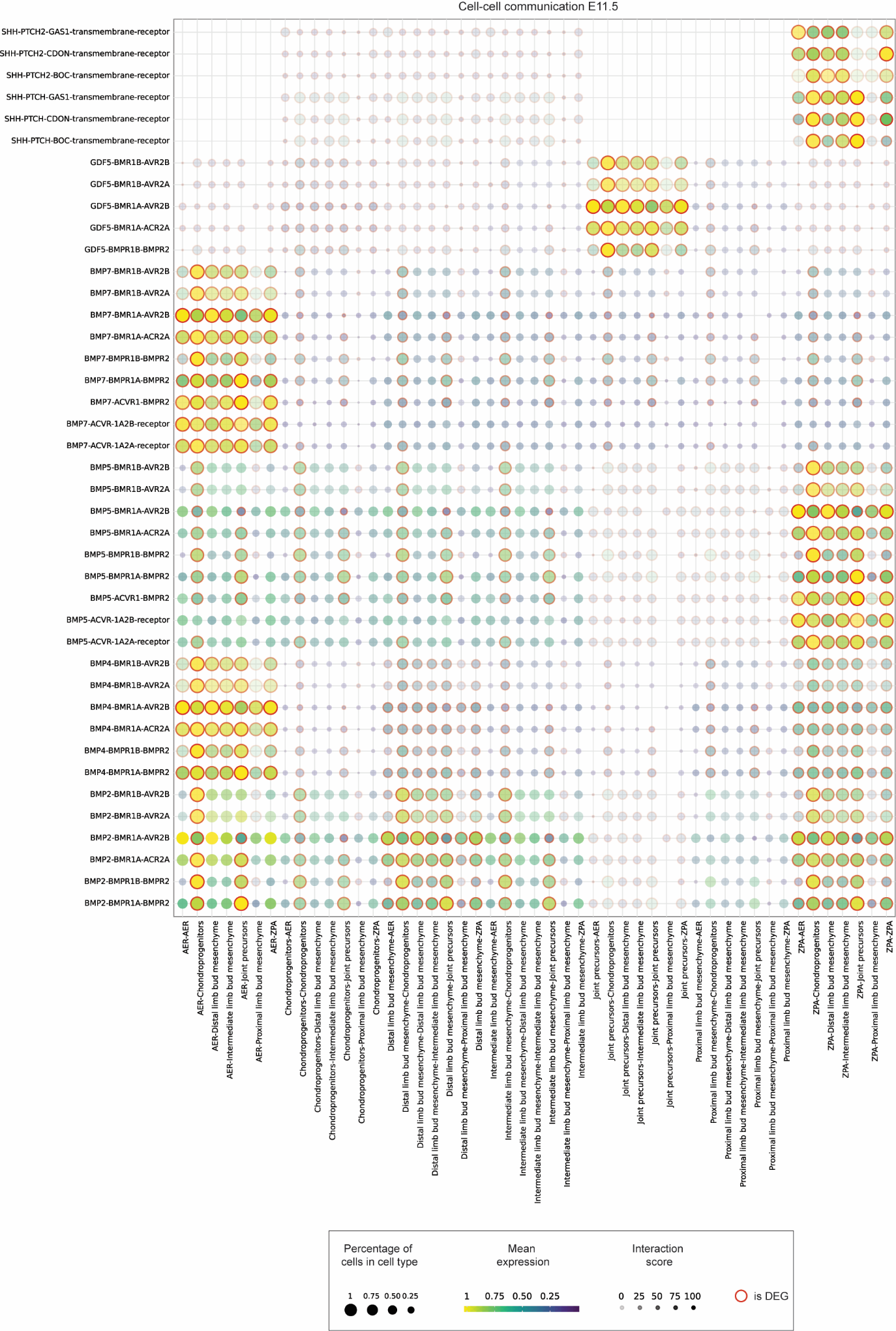


**Supplementary Figure 6. BMP, GDF5 and SHH signaling case study between AER, ZPA and mesenchyme for developmental timepoint E11.5.** Dot plot showing predicted ligand-receptor complex interactions at E11.5. The sender and receiver cell types are displayed on the x-axis, while ligand/receptor pairs are shown on the y-axis. The color intensity of each dot represents the mean gene expression, and the size indicates the percentage of cells expressing the gene within each cell type. Translucency reflects interaction specificity, and an outer red ring indicates differentially expressed genes (DEGs). Cell types: AER: Apical Ectodermal Ridge, PLBM: Proximal limb bud mesenchyme, ILBM: Intermediate limb bud mesenchyme, DLBM: Distal limb bud mesenchyme, CP: Chondroprogenitors, RZC: Resting zone chondrocytes, PC: Proliferative chondrocytes, PHC: Pre-hypertrophic chondrocytes, JP: Joint precursors.


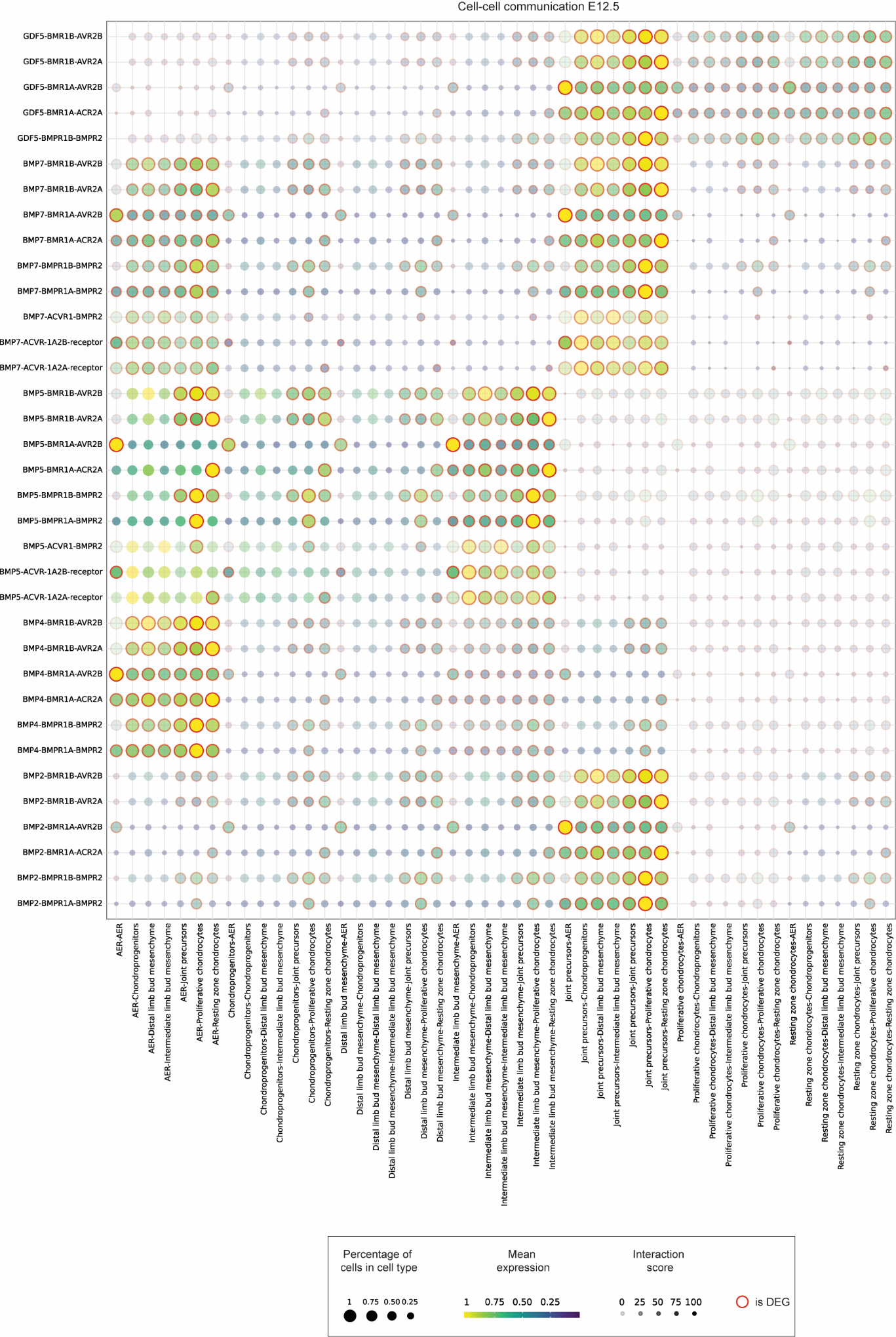


**Supplementary Figure 7. BMP, GDF5 and SHH signaling between AER and mesenchyme for developmental timepoint E12.5.** Dot plot showing predicted ligand-receptor complex interactions at E12.5. The sender and receiver cell types are displayed on the x-axis, while ligand/receptor pairs are shown on the y-axis. The color intensity of each dot represents the mean gene expression, and the size indicates the percentage of cells expressing the gene within each cell type. Translucency reflects interaction specificity, and an outer red ring indicates differentially expressed genes (DEGs). Cell types: AER: Apical Ectodermal Ridge, PLBM: Proximal limb bud mesenchyme, ILBM: Intermediate limb bud mesenchyme, DLBM: Distal limb bud mesenchyme, CP: Chondroprogenitors, RZC: Resting zone chondrocytes, PC: Proliferative chondrocytes, PHC: Pre-hypertrophic chondrocytes, JP: Joint precursors, ZPA: Zone of Polarizing Activity.

**
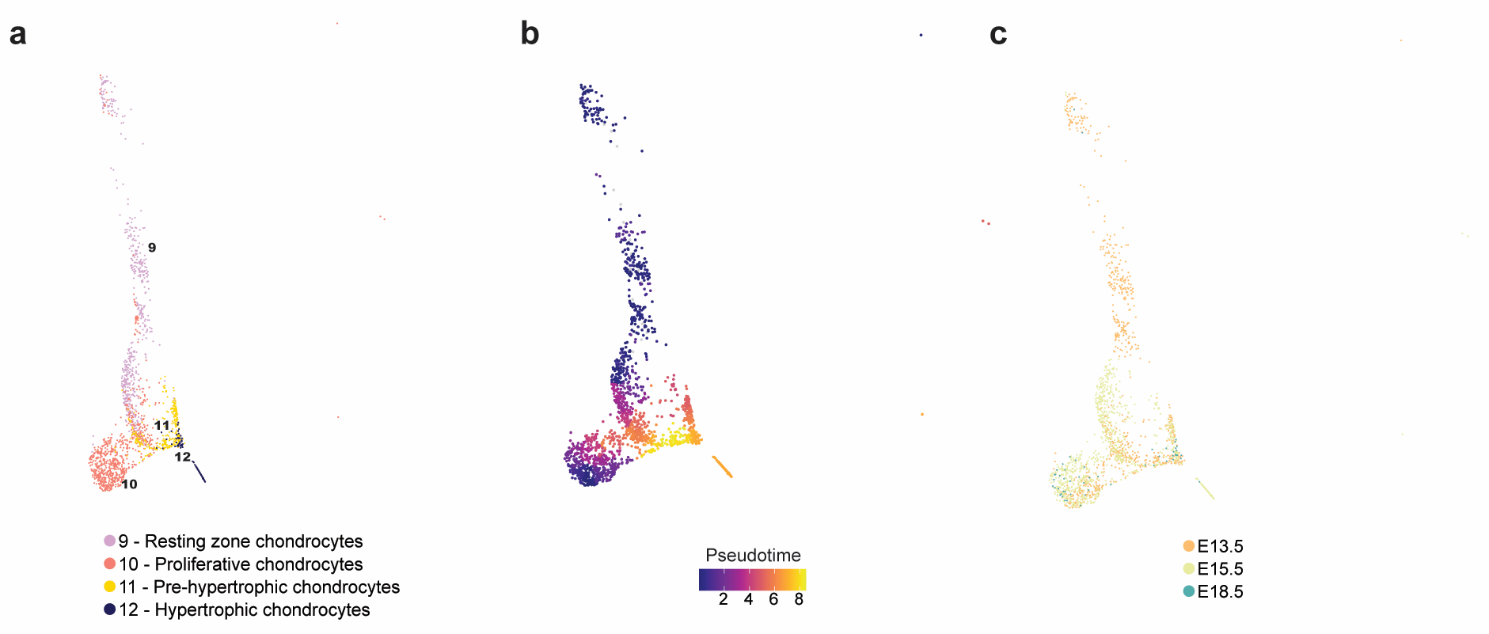
**

**Supplementary Figure 8. Monocle3 pseudotime analysis of growth plate cell types in the** **SRP238355 dataset.** UMAP visualization of the SRP238355 dataset, incorporating multiple time points across growth plate cell identities. The left panel shows cell states colored by annotation (**a)**, offering an overview of the different cell populations. The middle panel depicts the progression of the dataset along pseudotime (**b)**, as calculated using Monocle3, with cells colored according to their pseudotime values. The right panel shows the dataset colored by developmental time point (**c)**, highlighting the temporal dynamics of growth plate cell types during development.

**
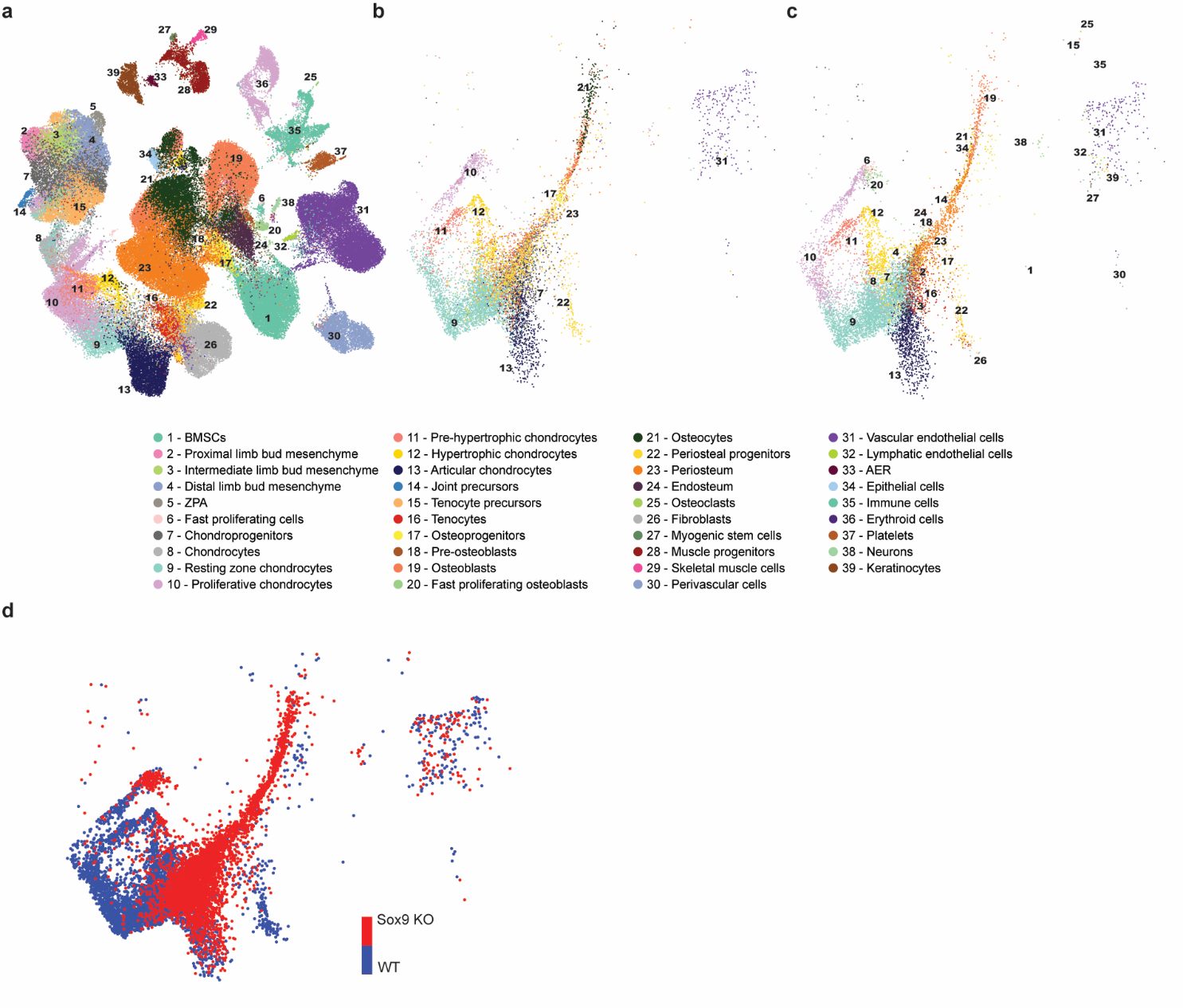
**

**Supplementary Figure 9. Ground truth wildtype and Sox9 knockout data projected on a subset of the LSCA. a**, UMAP visualization of a subset of the LSCA integrated with the WT and ground truth KO dataset (SRP293998) in the scANVI latent space. **b**, Ground truth dataset with manually labeled cell types. **c**, Ground truth dataset with automatically labeled cell types using scvi-tools. **d,** Integrated LSCA and ground truth dataset visualized by genotype, with red representing Sox9 knockout (KO) data and blue representing wild-type (WT) data.

**
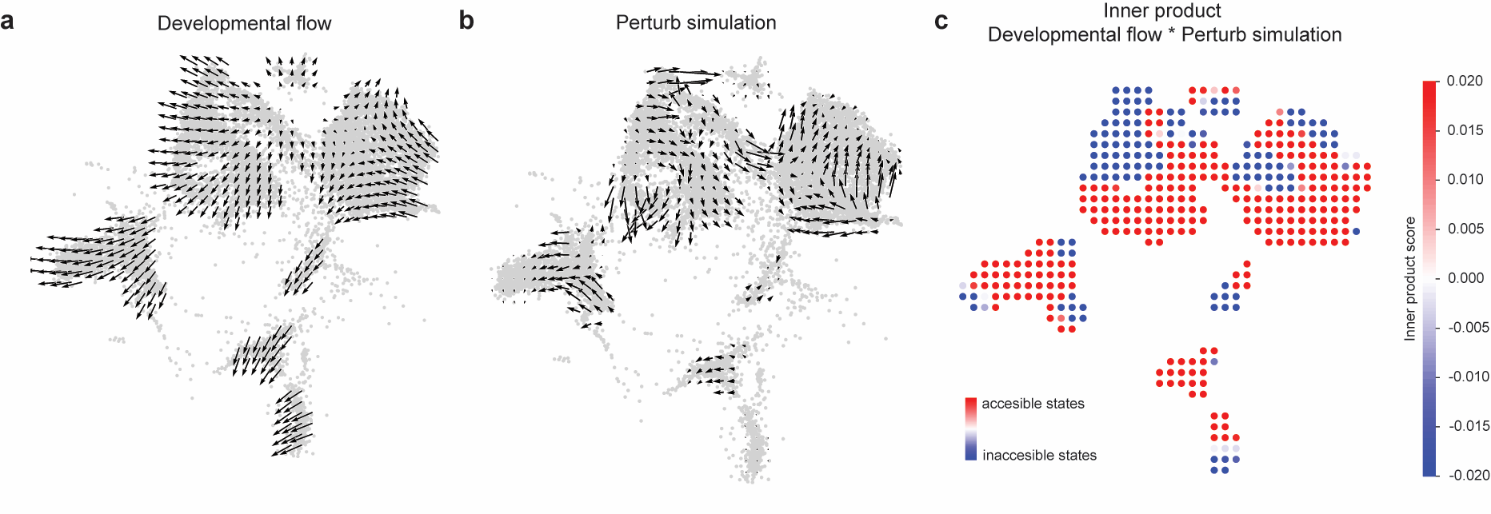
**

**Supplementary Figure 10**. **Sox9 knockout (KO*) in silico* predictions**. **a**, Gradient of pseudotime in the absence of perturbation. This gradient illustrates the progression of cells over time in a normal, unperturbed condition. **b**, CellOracle simulation of cell state transition vectors in a Sox9 KO condition. **c**, Inner vector product of vector fields shown in (**a**) and (**b**): the pseudotime gradient from the unperturbed condition and the cell state transition probability following *in silico* Sox9 perturbation. Red indicates the accessible states, defined as alignment of both vectors, while blue indicates the inaccessible states, defined as opposed vector directions.

| **Study (Accession)** | **Study (PMID)** | **Study (First, Last Author)** | **Technology** | **Developmental stage** | **Tissue origin** |
| --- | --- | --- | --- | --- | --- |
| PRJNA658566/ SRP278393 | 34260921 | Sivaraj, Adams | 10x | P21 | Bone marrow stromal cells |
| PRJNA870944/ SRP392940 | 36175067, 37873464 | Maerz, Farrell | 10x | 12-16w | Knee Synovium |
| PRJNA416038/ SRP122545 | 30250253 | Debnath, Landau & Greenblatt | CEL-seq2 | P7 | CTSK-mGFP+ cells from Femur |
| PRJNA597000/ SRP238355 | 31874220 | Kelly, Guilak | 10x | E11.5, E13.5, E15.5, E18,5 | Hindlimb |
| PRJNA666650/ SRP285918 | 33297480 | Desanlis, Kmita | 10x | E10.5, E11.5, E12.5 | Forelimb |
| PRJNA680336/ SRP293998 | 33597301 | Haseeb, Lefebvre | 10x | P13 | Tibial and Femur Epiphyses |
| PRJEB33444/ ERP116235 | 31543445 | Bohm, Maes | Smart-seq2 | E15.5 | Osx-Cre:GFP+ cells from Tibia and Humerus |
| PRJNA1080020/ SRP491509 | 38479598 | Nookaew, O’Brien | 10x | 8-14w | Periosteum, Endosteum |
| PRJNA527721/ SRP188674 | 31130381 | Baryawno, Regev & Scadden | 10x | 8-10w | Bone marrow stromal cells |
| PRJNA858479/ SRP386294 | 36777346 | Agoro, White | 10x | 8w | Cortical Bone |

**Supplementary Table 1**: scRNA-seq datasets used to construct the Limb Skeletal Cell Atlas. In this table, we list study accession numbers, PMID, first and last author, technology used, developmental stage, and origin of tissue.

| **Cell Type/ Cell State** | **Gene names** | **Gene ID** | **References** |
| --- | --- | --- | --- |
| BMSCs | Lepr, Cxcl12, Pdgfra, Pdgfrb | 16847, 20315, 18595, 18596 | ^1–3^ |
| Proximal limb bud mesenchyme | Prrx1, Hoxa9, Hoxd9, (Hoxa/d11-, Hoxa/d13-) | 18933, 15405, 15438, (15396, 15431) | ^4–6^ |
| Intermediate limb bud mesenchyme | Prrx1, Hoxa11, Hoxd11, (Hoxa/d13-) | 18933, 15396, 15431, (15398, 15433) | ^4–6^ |
| Proximal limb bud mesenchyme | Prrx1, Hoxa13, Hoxd13 | 18933, 15398, 15433 | ^4–6^ |
| ZPA | Prrx1, Prrx2, Shh | 18933, 20204, 20423 | ^6,7^ |
| Fast proliferating cells | Cdk1, Cdc20 | 12534, 107995 | ^8,9^ |
| Chondroprogenitors | Prrx1, Prrx2, Sox9 | 18933, 20204, 20682 | ^6^ |
| Chondrocytes | Sox9, Col2a1, Acan | 20682, 12824, 11595 | ^10^ |
| Resting zone chondrocytes | Col2a1, Ucma, Pthlh | 12824, 68527, 19227 | ^11,12^ |
| Proliferative chondrocytes | Col2a1, Top2a, Pth1r | 12824, 21973, 19228 | ^11^ |
| Pre-hypertrophic chondrocytes | Col2a1, Pth1r, Ihh | 12824, 19228, 16147 | ^11^ |
| Hypertrophic chondrocytes | Col2a1, Col10a1, Mmp13 | 12824, 12813, 17386 | ^11^ |
| Articular chondrocytes | Col2a1, Prg4 | 12824, 96875 | ^13^ |
| Joint precursors | Gdf5, Wnt9a, (Sox9-) | 14563, 216795, (20682) | ^14^ |
| Tenocyte precursors | Prrx1, Tnmd, Scx | 18933, 64103, 20289 | ^6,15^ |
| Tenocytes | Tnmd, Scx, (Prrx1-, Prrx2-) | 64103, 20289, (18933, 20204) | ^15^ |
| Osteoprogenitors | Prrx1, Runx2, Sp7, (Bglap-) | 18933, 12393, 170574, (12096) | ^16–18^ |
| Pre-osteoblasts | Runx2, Sp7, Spp1, (Prrx1-, Bglap-) | 12393, 170574, 20750, (18933,12096) | ^17–19^ |
| Osteoblasts | Runx2, Bglap, (Prrx1-) | 12393, 12096, (18933) | ^18,20^ |
| Fast proliferating osteoblasts | Cdk1, Cdc20, Bglap | 12534, 107995, 12096 | ^20–22^ |
| Osteocytes | Dmp1, Sost | 13406, 74499 | ^19,23^ |
| Periosteal progenitors | Prrx1, Prrx2, Postn, Ctsk | 18933, 20204, 50706, 13038 | ^19,24,25^ |
| Periosteum | Postn, Wnt16, Ctsk (Prrx1-, metadata original publication) | 50706, 93735, 13038, (18933) | ^19,25^ |
| Endosteum | Postn, Wnt16, Ctsk (Prrx1-, metadata original publication) | 50706, 93735, 13038, (18933) | ^19,25^ |
| Osteoclasts | Cd68, Ctsk, Ptprc | 12514, 13038, 19264 | ^26,27^ |
| Fibroblasts | S100a4, Dcn, Cilp | 20198, 13179, 214425 | ^28^ |
| Myogenic stem cells | Pax3, Pdgfc | 18505, 54635 | ^29,30^ |
| Muscle progenitors | Pax7, Itm2a | 18509, 16431 | ^29,30^ |
| Skeletal muscle cells | Myhc, Tnnt1, Tnnt3 | 111671, 21955, 21957 | ^30,31^ |
| Perivascular cells | Acta2, Rgs5 | 11475, 19737 | ^32^ |
| Vascular endothelial cells | Emcn, Pecam1, Cdh5, (Lyve1-) | 59308, 18613, 12562, (114332) | ^33^ |
| Lymphatic endothelial cells | Pecam1, Lyve1, Flt4 | 18613, 114332, 14257 | ^33^ |
| AER | Fgf8, Fgf4 | 14179, 14175 | ^34^ |
| Epithelial cells | Epcam | 17075 | ^33^ |
| Immune cells | Ptprc, Cd68, Camp | 19264, 12514, 12796 | ^33^ |
| Erythroid cells | Gypa, Car2 | 14934, 12349 | ^33^ |
| Platelets | Pf4, Vwf | 56744, 22371 | ^33^ |
| Neurons | Mpz, Ednrb | 17528, 13618 | ^33^ |
| Keratinocytes | Krt5, Krt10 | 110308, 16661 | ^33^ |

**Supplementary Table 2**: List of genes used to annotate clusters in the Limb Skeletal Cell Atlas. In this table, we list per cell type the genes used, gene IDs, and references.

**1** Tencerova, M. *et al.* *Frontiers in Endocrinology* 7, (2016) **2** Gilbert, W. *et al.* *Cytokine* 123, (2019) **3** Sivaraj, K. K. *et al.* *Cell Rep* 36, (2021) **4** Zakany, J. *et al.* *Current Opinion in Genetics and Development* 17, (2007) **5** Nelson, C. E. *et al.* *Development* 122, (1996) **6** Reinhardt, R. *et al.* *Development (Cambridge)* 146, (2019) **7** Tickle, C. *et al.* *Frontiers in Cell and Developmental Biology* 5, (2017) **8** Massacci, G. *et al.* *Br J Cancer* 129, (2023) **9** Bruno, S. *et al.* *Journal of Experimental and Clinical Cancer Research* 41, (2022) **10** Lefebvre, V. *et al.* *Connective Tissue Research* 58, (2017) **11** Kronenberg, H. M. *Nature* 423, (2003) **12** Tagariello, A. *et al.* *Matrix Biology* 27, (2008) **13** Kozhemyakina, E. *et al.* *Arthritis and Rheumatology* 67, (2015) **14** Roelofs, A. J. *et al.* *Nat Commun* 8, (2017) **15** Subramanian, A. *et al.* *Development (Cambridge)* 142, (2015) **16** Ouyang, Z. *et al.* *Bone* 58, (2014) **17** Rashid, H. *et al.* in *Connective Tissue Research* 55, (2014) **18** Komori, T. *International Journal of Molecular Sciences* 20, (2019) **19** Nookaew, I. *et al.* *Journal of Biological Chemistry* 300, (2024) **20** Zoch, M. L. *et al.* *Bone* 82, (2016) **21** Locard-Paulet, M. *et al.* *PLoS Comput Biol* 18, (2022) **22** Du, Y. *et al.* *EMBO Rep* 22, (2021) **23** Wee, N. K. *et al.* *Current Osteoporosis Reports* 19, (2021) **24** Brown, S. *et al.* *JBMR Plus* 7, (2023) **25** Debnath, S. *et al.* *Nature* 562, (2018) **26** Sun, Y. *et al.* *Frontiers in Immunology* 12, (2021) **27** Shalev, M. *et al.* *Biochimica et Biophysica Acta - Molecular Cell Research* 1866, (2019) **28** Fan, C. *et al.* *Front Genet* 13, (2022) **29** Buckingham, M. *et al.* *Seminars in Cell and Developmental Biology* 44, (2015) **30** Luo, H. *et al.* *Frontiers in Cell and Developmental Biology* 9, (2021) **31** Wei, B. *et al.* *Gene* 582, (2016) **32** Kumar, A. *et al.* *Cell Rep* 19, (2017) **33** Schaum, N. *et al.* *Nature* 562, (2018) **34** Lewandoski, M. *et al.* *Nat Genet* 26, (2000)
